# Enhancing the Reliability of NR-V2X Sidelink Broadcast Through Relaying and Dynamic Retransmission Control †

**DOI:** 10.3390/s26206392

**Published:** 2026-10-09

**Authors:** Neo Takahashi, Shiro Aoki, Suhua Tang

**Affiliations:** Graduate School of Informatics and Engineering, The University of Electro-Communications, Chofu 182-8585, Tokyo, Japan; n.takahashi@can.lab.uec.ac.jp (N.T.); s.aoki@can.lab.uec.ac.jp (S.A.)

**Keywords:** NR-V2X, sidelink, broadcast, relay, blind retransmission, channel busy ratio, congestion control

## Abstract

NR-V2X sidelink broadcast is an important communication mode for delivering safety-related information to surrounding vehicles with low latency. However, in non-line-of-sight (NLOS) environments such as intersections, direct links are blocked, and the blind retransmission mechanism defined for NR-V2X provides limited reliability improvement. This paper extends our previously reported relay-assisted retransmission scheme, RRT + ACK, which combines relay selection using link-quality indicators aggregated per direction (AvgLQIs/Dir) with ACK-assisted transfer of retransmission responsibility to the selected relay. The extension is a dynamic retransmission control (RT-Ctrl) method based on the Channel Busy Ratio (CBR), intended to limit retransmission-induced congestion. Simulations using Scenargie in NLOS intersection environments, under the assumption of ideal error-free ACK delivery, show that the basic RRT + ACK scheme improves the evaluation packet delivery ratio within 200 m (${\mathrm{EPDR}}_{200m}$) by approximately 8.6% compared with conventional blind retransmission. Under high vehicle density, the RT-Ctrl method with the best parameter setting improves ${\mathrm{EPDR}}_{200m}$ from 0.783 to 0.867 and reduces the average CBR from 0.594 to 0.329 compared with RRT + ACK using a fixed retransmission count.

## 1. Introduction

Vehicle-to-everything (V2X) communications, through which vehicles share information with nearby vehicles and infrastructure, are becoming increasingly important for improving road safety and advancing automated driving. In particular, sidelink communication enables vehicles to communicate directly without base-station assistance and is used to periodically disseminate safety information such as position, speed, acceleration, and heading to surrounding vehicles.

The 3rd Generation Partnership Project (3GPP) standardized LTE-based LTE-V2X in Release 14 and subsequently specified 5G New Radio (NR)-based NR-V2X sidelink in Release 16 [1,2,3]. NR-V2X sidelink defines Mode 1, in which a base station allocates radio resources, and Mode 2, in which each vehicle autonomously selects resources in a distributed manner [3,4]. Because Mode 2 uses sensing-based autonomous resource selection, it can operate outside base-station coverage [5]. However, reception reliability degrades under high vehicle density or in the presence of obstacles because of transmission collisions, half-duplex constraints, and non-line-of-sight (NLOS) propagation [5,6,7].

NR-V2X sidelink broadcast uses blind retransmission, in which a packet is retransmitted a predetermined number of times, to improve reliability [3]. When the direct link between a source vehicle and receiving vehicles is obstructed by buildings at an intersection, however, repeated transmissions from the same source remain affected by the same blockage [7,8]. Consequently, blind retransmission provides only limited improvement in an NLOS environment. Relaying through another vehicle is effective in such situations because it provides a different propagation path.

Relaying and retransmission nevertheless increase the number of transmissions and intensify contention for radio resources. In particular, using a fixed retransmission count in a high-density environment may aggravate channel congestion and even reduce the packet delivery ratio. A practical design must therefore both mitigate NLOS propagation through relaying and suppress unnecessary retransmissions when the channel is congested.

Our APCC 2024 paper introduced directionally aggregated LQIs and ACK-assisted transfer of retransmission responsibility, together with an evaluation using 40 vehicles and one retransmission [9]. These components form the basic RRT + ACK scheme retained here and are not new contributions of this article. The APCC paper identified congestion-dependent retransmission-count adjustment as future work. This article develops and evaluates a specific controller for that problem, and addresses the tradeoff between reliability and channel load when that relay mechanism is used with multiple retransmissions at higher vehicle density. Specifically, it introduces CBR-based control of both a session-level retransmission limit and a probabilistically selected retransmission count at SPS resource-reselection boundaries. It also extends the evaluation to 80 vehicles, compares fixed retransmission counts of one and four, and examines three controller parameter settings. Therefore, the additional contribution is congestion-aware retransmission control in the relay mechanism and its evaluation.

The remainder of this paper is organized as follows. Section 2 reviews NR-V2X sidelink and its technical challenges. Section 3 discusses related work and positions the present study. Section 4 describes the proposed schemes. Section 5 presents the simulation evaluation and discussion. Finally, Section 6 concludes the paper and outlines future work.

## 2. Overview of C-V2X Sidelink

### 2.1. C-V2X

Cellular Vehicle-to-Everything (C-V2X) connects vehicles with nearby vehicles, pedestrians, roadside units, and networks using cellular communication technologies [10]. 3GPP specified LTE-based LTE-V2X in Release 14 and 5G NR-based NR-V2X in Release 16 [1,2,3]. NR-V2X complements rather than replaces LTE-V2X and supports more advanced V2X use cases [3].

C-V2X defines cellular uplink and downlink for wide-area communications through a base station, as well as sidelink for direct communication between user equipments. Uplink carries traffic from vehicles to the base station, whereas downlink carries traffic from the base station to vehicles. Sidelink, by contrast, supports direct communication without routing packets through a base station and is therefore used to share safety information among nearby vehicles.

### 2.2. Sidelink Radio Resources

In V2X communications, packets are transmitted using radio resources organized in the time and frequency domains. LTE-V2X uses subframes as its time unit, whereas NR-V2X uses slots. Sidelink transmissions use the Physical Sidelink Control Channel (PSCCH) for control information and the Physical Sidelink Shared Channel (PSSCH) for data [3].

The PSCCH carries Sidelink Control Information (SCI), including reservation information of the resources used for future transmission. The PSSCH carries data such as vehicle position, speed, acceleration, and heading. By combining control information and data, a receiving vehicle can determine the transmission resources and decode the associated packet.

### 2.3. NR-V2X Sidelink

To support more advanced V2X use cases than LTE-V2X, NR-V2X sidelink supports unicast, groupcast, and broadcast [3,4]. It also introduces Hybrid Automatic Repeat reQuest (HARQ) retransmission and the Physical Sidelink Feedback Channel (PSFCH) for feedback [3,4].

In NR-V2X, the PSCCH carries the first-stage SCI, while the PSSCH carries the second-stage SCI and data. First-stage SCI includes information about PSSCH resource reservations. Second-stage SCI includes additional information required for decoding, such as HARQ-related information and the New Data Indicator (NDI), which identifies whether the transmission is an initial transmission or a retransmission.

NR-V2X sidelink defines Mode 1 and Mode 2 resource allocation [3,4]. Mode 1 corresponds to LTE-V2X Mode 3 and uses base stations for resource allocation. Mode 2 corresponds to LTE-V2X Mode 4 and allows each vehicle to autonomously select resources using carrier sensing. This study focuses on NR-V2X sidelink Mode 2, which can operate without base-station assistance.

### 2.4. Blind Retransmission

Retransmission is effective against temporary link-quality degradation caused by transmission collisions or channel fading [3]. In unicast and some groupcast configurations, the transmitter can determine whether retransmission is necessary from an ACK or NACK sent by the receiver. In broadcast, however, the destination is not limited to one vehicle, and a packet is intended for many nearby vehicles. Collecting reception results from every receiving vehicle is therefore impractical. NR-V2X sidelink broadcast consequently uses blind retransmission, which retransmits a packet a predetermined number of times irrespective of the reception status.

LTE-V2X permits only one blind retransmission, whereas NR-V2X permits up to $N_{\mathrm{tx},\max}-1$ blind retransmissions [3]. Here, $N_{\mathrm{tx},\max}$ denotes the maximum number of transmission opportunities reserved for one packet and is configured within $1\;\leq \;N_{\mathrm{tx},\max}\;\leq \;32$. When $N_{\mathrm{tx},\max}=1$, only the initial transmission is performed; when $N_{\mathrm{tx},\max}>1$, at most $N_{\mathrm{tx},\max}-1$ retransmissions follow the initial transmission. Although $N_{\mathrm{tx},\max}$ is preconfigured, it may be changed by congestion control according to channel usage.

First-stage SCI announces the resource reservations used by NR-V2X sidelink transmissions. The reservation range is constrained such that only resources within a window *W* of 32 slots beginning with the transmitting slot can be announced. Furthermore, one first-stage SCI can announce only $N_{\mathrm{SCI}}$ resources, where $2\leq N_{\mathrm{SCI}}\leq 3$. Reserving $N_{\mathrm{tx},\max}$ transmission resources may therefore require multiple first-stage SCI messages for each window *W*.

NDI distinguishes the initial transmission from retransmissions in each transmission resource. It is set to 1 for the initial transmission resource and to 0 for subsequent retransmission resources, thereby enabling receiving vehicles to distinguish new data from retransmitted data.

### 2.5. Semi-Persistent Scheduling

NR-V2X sidelink Mode 2 uses Semi-Persistent Scheduling (SPS) for periodic transmissions [3,5,6]. Each vehicle observes the received signal strength and the reservations announced by other vehicles within a sensing window that covers past radio resources. Based on the sensing result, each vehicle selects its transmission resource from a future selection window.

Each vehicle continues to use a selected resource for a specified number of transmissions. A reselection counter records the remaining number of uses, and each vehicle selects a resource again from the sensing result when the counter reaches zero. SPS thereby stabilizes resource selection for periodic safety-message dissemination.

### 2.6. Distributed Congestion Control in C-V2X

Because many vehicles share the same resource pool in C-V2X, the Channel Busy Ratio (CBR) and Channel Occupancy Ratio (CR) are used to characterize congestion [11,12]. CBR is the proportion of resources classified as busy within an observation window and thus indicates overall channel congestion. CR represents the amount of radio resources used by the local user equipment in the past and scheduled for its use in the near future; it therefore characterizes the local channel occupancy.

Distributed congestion control in C-V2X sets an allowable CR limit according to the observed CBR and can adjust parameters such as the transmission interval, number of occupied subchannels, modulation and coding scheme (MCS), and transmission power [12]. In this study, the retransmission-count controller described in Section 4.3 uses CBR as its channel-congestion indicator.

## 3. Related Work

### 3.1. Relaying for Unicast

Relaying in V2X communications has been widely studied as a means of supplementing a poor direct link between a source and a destination through a third vehicle or base station. In unicast, the principal objective is to improve the link quality for a particular source-–receiver pair. Existing proposals include cooperative relaying that considers interference and outdated channel-state information [13], as well as relay selection based on multiple quality metrics [14]. Reliability analyses have also considered cooperative C-V2X that combines direct and relay paths, and two-hop transmission has been studied under blockage by large vehicles [15,16]. For NR-V2X sidelink, Reference Signal Received Power (RSRP) has been investigated as a relay-candidate selection criterion [17].

Unicast relaying needs to improve the link to a particular receiving vehicle, whereas broadcast must simultaneously deliver information to multiple nearby vehicles. A relay-selection method designed for unicast therefore cannot be directly applied to sidelink broadcast.

### 3.2. Relaying for Broadcast

Broadcast safety information is intended for many vehicles surrounding the source rather than for one specified receiver. Relay selection must therefore increase the packet delivery ratio over the entire set of surrounding vehicles, rather than optimize only one source–receiver link.

For Dedicated Short-Range Communications (DSRC), using a roadside unit as a relay has been shown to improve safety-message broadcast reliability at intersections in urban environments [18]. Comparative and hybrid studies of DSRC and C-V2X have also clarified their different latency and reliability characteristics [19,20]. Roadside-unit relaying, however, requires infrastructure deployment and is therefore available only in limited areas. Network-layer multihop broadcast has also been extensively studied [21], including schemes intended to extend the dissemination range and improve the reliability of emergency safety messages [22,23].

The performance of broadcast transmission degrades greatly in the NLOS environment [7,8], due to the obstruction of the line-of-sight path, and the lack of feedback on transmission failure. Relaying provides a potential alternative propagation path when the direct link is obstructed, motivating its use for reliable broadcast at NLOS intersections [9,18,24]. The reservation-reuse approach considered here follows the NR-V2X broadcast relay mechanism in [24]: a selected relay uses retransmission resourceresources already reserved by the source, aiming to achieve the same latency as the retransmission over the direct link. This design targets local delivery around the source, rather than dissemination through an arbitrary chain of forwarding vehicles. Reusing resource reservations avoids a separate resource-selection procedure for the relay, but does not by itself eliminate forwarding, processing, or feedback delay. Its suitability for a particular service therefore also depends on that service’s delivery deadline.

### 3.3. Distributed Resource Allocation and Collisions

In NR-V2X sidelink Mode 2, each vehicle autonomously selects resources without base-station assistance. Although this operation supports out-of-coverage communication, many vehicles share the same resource pool, and transmission collisions, the hidden-terminal problem, and half-duplex constraints can reduce packet reception [5].

An evaluation of sidelink broadcast reliability must account not only for failures caused by radio propagation but also for MAC-layer resource contention and transmission collisions [5,6]. Additional retransmissions and relaying increase the number of reception opportunities, but they also increase the number of transmissions and may therefore raise resource contention and the collision probability. Related solutions to this problem include full-duplex collision detection, region-based coordination for distributed resource allocation, and improved time-domain resource allocation for NR-V2X sidelink [25,26,27].

These studies mainly address resource allocation and collision suppression but do not sufficiently investigate how channel congestion should govern the additional traffic generated by relaying and retransmission. A relay-assisted scheme must therefore be evaluated not only for its propagation gain at the physical layer but also for its impact on resource contention under distributed Mode 2 resource allocation.

### 3.4. Congestion Control

Increasing the retransmission count can improve reliability but can also increase channel load, collisions, and interference. Under congestion, the transmitted traffic should therefore be adjusted to the channel condition instead of simply increasing retransmissions.

Comparisons of C-V2X with and without blind retransmission have shown that retransmission may increase channel load and worsen reception performance in high-density environments [28]. Adaptive retransmission is not, by itself, a new concept in NR-V2X. An adaptive method has been proposed to control the number of blind retransmissions in NR-V2X Mode 2 according to the surrounding congestion [29], and is relevant to assessing alternatives to fixed retransmission counts. Other congestion-control approaches use CBR as a congestion indicator and reduce the transmission frequency by extending the transmission interval [30]. Xu et al. investigated velocity-adaptive access for semantic-aware NR-V2X Mode 2 networks, considering access fairness and Age of Information in vehicle–roadside-unit communication [31]. Their selection-window adaptation addresses differences in communication opportunities associated with vehicle speed. This is related to the need to account for mobility and information freshness, but has a different objective from the present local broadcast relay scheme, which controls retransmissions to balance NLOS delivery reliability against channel load.

These findings demonstrate that suppressing traffic under congestion can improve communication performance. In relay-assisted retransmission, however, the combined transmissions of the source and relay may increase channel occupancy. A framework is therefore needed to dynamically control the retransmission count for each vehicle while accounting for the congestion caused by relay-assisted retransmission traffic.

### 3.5. Prior Relay-Assisted Retransmission Scheme

The work directly underlying this study added relaying to the blind retransmission procedure specified for NR-V2X [24]. In that scheme, a relay vehicle *r* uses the retransmission resources reserved by a source vehicle *n* and retransmits the packet received from *n* on behalf of the source. Because the relay does not need to reserve new retransmission resources, relaying can be performed without the additional latency associated with a new resource reservation.

The prior scheme uses the Packet Reception Ratio (PRR) as a Link Quality Indicator (LQI). Let $p_{n,r}$ denote the PRR from source vehicle *n* to relay candidate *r*, and let $p_{r,i}$ denote the PRR from *r* to receiving vehicle *i*. When *r* performs *K* relay retransmissions, the probability $p_{n,r,i}$ that the transmission from *n* reaches *r* and that *i* subsequently receives at least one relay transmission is

(1) $$p_{n,r,i}=p_{n,r}\left\{1-{\left(1-p_{r,i}\right)}^{K}\right\}.$$

The combined probability $p_{n,i}^{r}$ that *i* successfully receives either the initial direct transmission from *n* or a relay retransmission from *r* is

(2) $$p_{n,i}^{r}=1-\left(1-p_{n,i}\right)\left(1-p_{n,r,i}\right).$$

Using this probability, the PDR $P_{n}^{r}$ from *n* to the set of its neighboring vehicles $N_{n}$ when *r* is used as the relay is calculated as

(3) $$P_{n}^{r}=\frac{1}{|N_{n}|}\left(p_{n,r}+\sum_{i\in N_{n},i\neq r}p_{n,i}^{r}\right).$$

The relay candidate that maximizes Equation (3) is selected as relay vehicle $r^{*}$:

(4) $$r^{*}=\arg\max_{r}P_{n}^{r}.$$

The above relay scheme [24] uses individual link-quality information for relay selection. Our subsequent APCC 2024 work [9] reduced the advertised LQI information through directional aggregation and introduced an explicit ACK to confirm the correct reception at the relay node. Its simulation evaluation used 40 vehicles and one fixed retransmission. That paper identified congestion-dependent retransmission-count adjustment as future work. The present article addresses this remaining issue by introducing CBR-based retransmission control and examining the tradeoff between delivery rate and channel load under the expanded evaluation conditions.

### 3.6. Research Gaps

The question addressed here is how to control retransmission traffic when relay selection and explicit ACK feedback are combined with autonomous Mode 2 resource allocation. The reservation-reuse mechanism was introduced in [24], and directional LQI aggregation and ACK-assisted transfer of retransmission responsibility were subsequently studied in [9]. These earlier components address alternative propagation paths, information exchange, and source-to-relay reception failure. They do not by themselves determine how many retransmissions should be retained as channel load increases. Motivated by the resource-contention impacts identified in Mode 2 studies [5,6] and the relevance of adaptive retransmission [29], this article examines CBR-based retransmission control in the relay framework. The objective is to retain useful additional reception opportunities while limiting the traffic generated by repeated source and relay transmissions.

## 4. Proposed Schemes

The basic RRT + ACK scheme, previously reported in [9], combines directional link-quality information with ACK-assisted transfer of retransmission responsibility. It reduces the number of LQI values to be shared and lets the source continue retransmitting when the selected relay has not received the packet. The extension proposed in this article, RT-Ctrl, applies CBR-based retransmission control to this mechanism to limit excessive retransmissions under congestion.

In the following, *n* denotes a source vehicle, *r* is a relay candidate, *i* is a receiving vehicle, and $N_{n}$ is the set of neighboring vehicles of *n*. The link quality between *n* and *i* is denoted by $p_{n,i}$, and *K* is the number of retransmissions.

### 4.1. Relay Selection Using Link Quality per Direction

As shown in Equation (1), the prior scheme uses the individual link quality $p_{r,i}$ from relay candidate *r* to each receiving vehicle *i* [24]. Selecting a relay consequently requires LQIs for every pair of vehicles to be shared. In the proposed scheme, each vehicle classifies its neighbors into the four directions North, South, East, and West and exchanges the Average LQIs per Direction (AvgLQIs/Dir). Let $N_{n,Dir}$ be the set of neighboring vehicles of source *n* classified into direction Dir. The average directional PRR is

(5) $$p_{n,Dir}=\frac{1}{|N_{n,Dir}|}\sum_{i\in N_{n,Dir}}p_{n,i}.$$

Suppose that relay candidate *r* performs *K* relay retransmissions. Let Dir(r,i) denote the direction of receiving vehicle *i* as viewed from *r*, and let $p_{r,Dir(r,i)}$ be the average PRR from *r* in that direction. The proposed scheme replaces $p_{r,i}$ in Equation (1) with $p_{r,Dir(r,i)}$ and approximates the probability that *i* receives a relay retransmission after the transmission from *n* to *r* succeeds as

(6) $$p_{n,r,i}=p_{n,r}\left\{1-{\left(1-p_{r,Dir(r,i)}\right)}^{K}\right\}.$$

As in the prior scheme, Equation (2) gives the success probability combining the initial direct transmission with the relay retransmissions, and Equations (3) and (4) select the relay vehicle $r^{*}$ that maximizes $P_{n}^{r}$. Using the directional average bounds the number of LQI values in each vehicle’s advertisement, and helps to reduce the control message size.

### 4.2. Transfer of Retransmission Responsibility Through an Explicit ACK

The selected relay can retransmit a packet only after receiving it from source *n*. In RRT + ACK, it returns an explicit ACK upon successful reception. Until the ACK is received, the source retains retransmission responsibility within the available retransmission budget; the relay then uses the remaining budget to continue retransmission. The data packet is intended for broadcast reception by surrounding vehicles. The ACK confirms reception at the selected relay only and does not acknowledge delivery to all neighboring receivers.

The selected relay acts as a representative receiver for coordination feedback, while the data packet remains broadcast. In the proposed design, this ACK is envisaged as groupcast-like feedback conveyed through PSFCH. The simulations, however, assume ideal error-free ACK delivery. The results therefore provide an ideal-feedback reference and do not quantify sensitivity to ACK loss or feedback transmission delay. Standardized sidelink HARQ feedback on PSFCH is specified for unicast and groupcast [32]; the proposed coordination role does not itself establish a standards-compliant broadcast-feedback implementation. Missing or delayed ACKs can delay transfer of retransmission responsibility. Repeated ACK transmission and timeout-based fallback are possible countermeasures, but their effectiveness and resource costs are not evaluated here.

### 4.3. CBR-Based Retransmission-Count Control

Under high vehicle density, increasing the retransmission count can aggravate channel congestion and consequently reduce reliability [28,29]. The proposed RT-Ctrl scheme treats SPS resource reselection as a session boundary. From the CBR observed at the beginning of each session, it determines both the maximum retransmission count $K_{\max}$ allowed during that session and the retransmission count $K_{\mathrm{retx}}$ that is actually performed [11,12].

The update of retransmission-count control is thus event-driven by SPS resource reselection, rather than performed for every physical-layer slot. Both retransmission -count values remain fixed during the session. This timing should be distinguished from the collection of reception statistics and the refresh of directional LQI information used for relay selection. The packet-generation interval alone does not specify the age of those link estimates. Holding the counts fixed avoids repeated changes to the session resource budget, but also delays adaptation to congestion changes occurring between reselection events.

Let $N_{\max}$ be the maximum retransmission count permitted by the scenario. Unlike $N_{\mathrm{tx},\max}$ in Section 2.4, $N_{\max}$ excludes the initial transmission and counts retransmissions only. The controller updates $K_{\max}$ using the target congestion level $T_{\mathrm{target}}$, severe-congestion threshold $T_{\max}$, deadband width *H*, and limit $K_{\max}^{\mathrm{prev}}$ used in the immediately preceding SPS session:

(7) $$K_{\max}=\left\{\begin{array}{cc} 1, & \mathrm{CBR}\geq T_{\max},\\ \max(K_{\max}^{\mathrm{prev}}-1,1), & T_{\mathrm{target}}+H<\mathrm{CBR}<T_{\max},\\ \min(K_{\max}^{\mathrm{prev}}+1,N_{\max}), & \mathrm{CBR}<T_{\mathrm{target}}-H,\\ K_{\max}^{\mathrm{prev}}, & \mathrm{otherwise}. \end{array}\right.$$

The first SPS session has no preceding session. Each vehicle therefore initializes $K_{\max}^{\mathrm{prev}}$ from a discrete uniform distribution over the integers from 1 to $N_{\max}$:

(8) $$\mathrm{Pr}\left(K_{\max}^{\mathrm{prev}}=k\right)=\frac{1}{N_{\max}},\;k\in \left\{1,2,\ldots ,N_{\max}\right\}.$$

This random initialization prevents all vehicles from beginning with the same control value, thereby reducing synchronization of their retransmission behavior and suppressing a burst of excessive retransmissions immediately after the session begins.

The initial transmission is always performed and is not included in $K_{\mathrm{retx}}$. For each of the $K_{\max}$ possible retransmission opportunities, a retransmission is selected with probability

(9) $$p_{\mathrm{retx}}\left(\mathrm{CBR}\right)=\left\{\begin{array}{cc} 0, & \mathrm{CBR}\geq T_{\max},\\ 1, & \mathrm{CBR}\leq T_{\mathrm{target}},\\ 1-\frac{\mathrm{CBR}-T_{\mathrm{target}}}{T_{\max}-T_{\mathrm{target}}},\; & T_{\mathrm{target}}<\mathrm{CBR}<T_{\max}. \end{array}\right.$$

The actual number of retransmissions is sampled as

(10) $$K_{\mathrm{retx}}∼\mathrm{Binomial}\left(K_{\max},p_{\mathrm{retx}}\left(\mathrm{CBR}\right)\right).$$

This gives $0\leq K_{\mathrm{retx}}\leq K_{\max}$ and $E\left[K_{\mathrm{retx}}\right]=K_{\max}p_{\mathrm{retx}}\left(\mathrm{CBR}\right)$. The total number of transmissions per packet, including the initial transmission, is $N_{\mathrm{tx}}=1+K_{\mathrm{retx}}$. Therefore, when $\mathrm{CBR}\geq T_{\max}$, $p_{\mathrm{retx}}=0$ and $K_{\mathrm{retx}}=0$, so only the initial transmission is performed.

**Figure 1:**
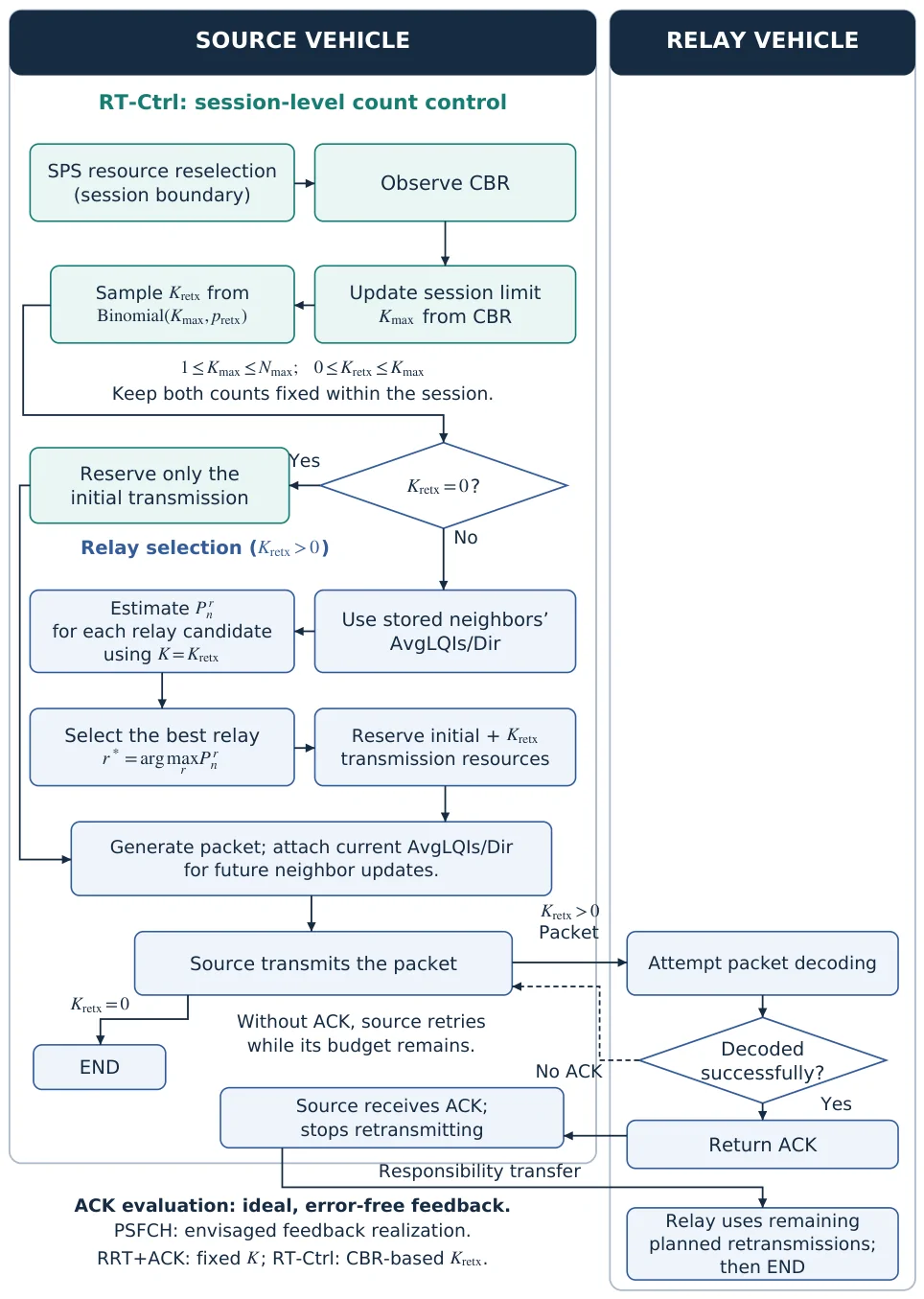
Framework of the basic RRT + ACK scheme and the extended RT-Ctrl scheme.

Figure 1 separates retransmission-count control at an SPS session boundary from packet transmission by the source and the selected relay. The source first observes CBR and determines the session retransmission count. A zero count leaves only the initial transmission; otherwise, the flow includes relay selection and reservation of retransmission opportunities. For each packet, receiving an ACK from the relay transfers responsibility for the remaining retransmissions from the source to the relay. The RRT + ACK baseline (fixed retransmission count) and the RT-Ctrl extension share this relay operation. The PSFCH and transfer annotations describe the intended coordination mechanism; the evaluation uses the ideal-feedback assumption stated above.

## 5. Simulation Evaluation

### 5.1. Simulation Environment

The simulation conditions are summarized in Table 1.

The evaluation builds on the Scenargie-based NR-V2X simulation framework used in our APCC 2024 study [9,33], with the retransmission-count controller added for the present evaluation. The evaluation uses an intersection model, representative of an NLOS environment, with buildings adjacent to the roads acting as obstacles. Vehicles move throughout each simulation. They generate packets every 100 ms for 100 s, from simulation time 10 s to 110 s. Packet generation is asynchronous among vehicles, with a maximum initial jitter of 100 ms.

Scenargie is a commercial network simulator widely used in Japan. It has the C-V2X function and technical support from the developers. The evaluation assumes ideal error-free ACK delivery and does not separately evaluate ACK loss, feedback transmission delay, and the feedback resource consumption. Accordingly, the reported results characterize the relay and retransmission-control mechanisms under this feedback assumption.

For the dynamic-control evaluation with 80 vehicles, the scenario-level upper bound on the retransmission count is set to $N_{\max}=4$. Therefore, $0\leq K_{\mathrm{retx}}\leq K_{\max}\leq N_{\max}=4$. Because the initial transmission is always performed, the total number of transmissions per packet ranges from 1 to 5. RT-Ctrl is evaluated using three parameter triples $\left(T_{\mathrm{target}},T_{\max},H\right)$: (0.20,0.40,0.02), (0.25,0.45,0.02), and (0.30,0.50,0.02).

### 5.2. Evaluation Metrics and Comparison Schemes

Each condition comprises 20 independent simulation runs. We first compute a metric within each run and then report the unweighted average of the 20 runs, the standard deviation (SD), and the two-sided 95% Student-*t* confidence interval $\bar{x}\pm t_{0.975,19}s/\sqrt{20}$, where $t_{0.975,19}=2.0930$. The statistical unit is one simulation run. These intervals describe uncertainty under the evaluation conditions.

As the reliability metric, the evaluation packet delivery ratio ${\mathrm{EPDR}}_{d}$ is defined as the proportion of vehicles that receive the same packet among the set $N_{d}$ located within radius *d* of the source vehicle. Within a run, EPDR is the arithmetic average of the per-packet reception ratios, excluding entries with no eligible receiver. The implementation counts receptions with delay below 100 ms, the deadline of local periodicalperiodic dissemination; this accounting window does not establish performance for other service deadlines. Channel congestion is evaluated from the CBR observed by the source at the initial transmission of each packet. Mean CBR is the arithmetic average of valid CBR observations recorded at the initial-transmission resource assignment of each packet. Each observation uses the preceding 100 slots (100 ms in this configuration); this measurement window is distinct from the run-level averaging period. To examine the controller behavior, we also evaluate the retransmission count. In the figure legends, ${\mathrm{RT}}_{\mathrm{num}}$ denotes a fixed retransmission count, whereas ${\mathrm{RT}}_{\max}$ denotes the scenario-level $N_{\max}$ used by dynamic control. Transmission counts are aggregated over the 20 simulation runs.

The basic schemes compared are conventional blind retransmission (BRT), relay-assisted retransmission using directional LQIs (RRT), and RRT with an explicit ACK (RRT + ACK). For the extended evaluation, RRT + ACK is used as the baseline and compared with CBR-based retransmission-count control (RT-Ctrl).

### 5.3. Evaluation of the Basic Scheme

**Figure 2:**
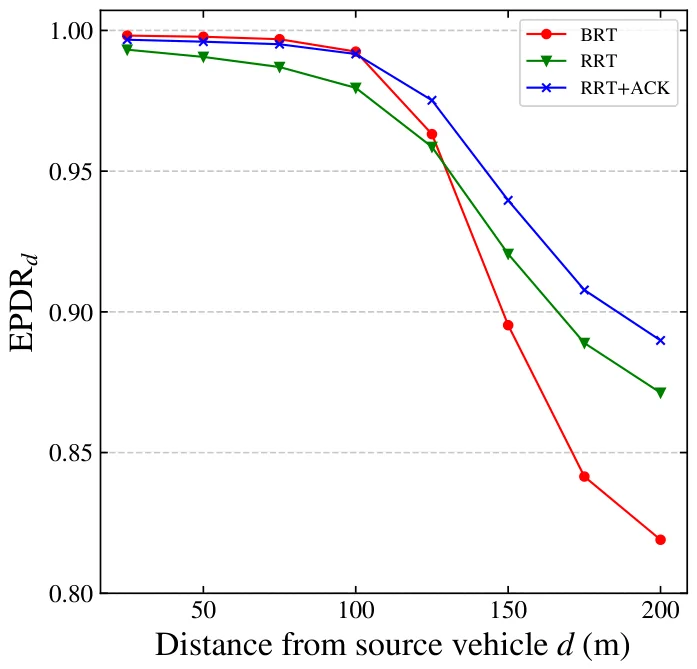
${\mathrm{EPDR}}_{d}$ with 40 vehicles and one retransmission.

We first consider a low-to-moderate-density scenario with 40 vehicles and one retransmission. Figure 2 plots ${\mathrm{EPDR}}_{d}$ at 25 m intervals. Table 2 and Table 3 report run-level variability. Appendix A tabulates the plotted distance values and selected CBR cumulative probabilities.

Compared with conventional BRT, RRT + ACK improves ${\mathrm{EPDR}}_{d}$ at distances greater than 125 m. The approximately 8.6% relative improvement in ${\mathrm{EPDR}}_{200m}$ is the observed benefit of the evaluated scheme combining directional-LQI-based relay selection with ACK-assisted retransmission coordination; this comparison does not isolate the contribution of directional LQI aggregation alone.

At 200 m, the ${\mathrm{EPDR}}_{d}$ increases from 0.8190 for BRT to 0.8899 for RRT + ACK, corresponding to a 0.0708 absolute gain and an 8.65% relative gain. Their 95% mean confidence intervals are [0.8177, 0.8203] and [0.8889, 0.8908], respectively. The separation is large relative to the observed run-to-run variability in this scenario. Equivalently, if we look at the packet loss rate, defined as $1.0-{\mathrm{EPDR}}_{d}$, it is decreased from 18.10% to 11.01%, with a relative reduction of approximately 39.1%. At short distances, RRT may have lower reliability than BRT because a failure onof the source-to-relay transmission prevents subsequent relay retransmissions. In RRT + ACK, the source continues retransmitting until the relay correctly receives the packet, suppressing this degradation.

**Figure 3:**
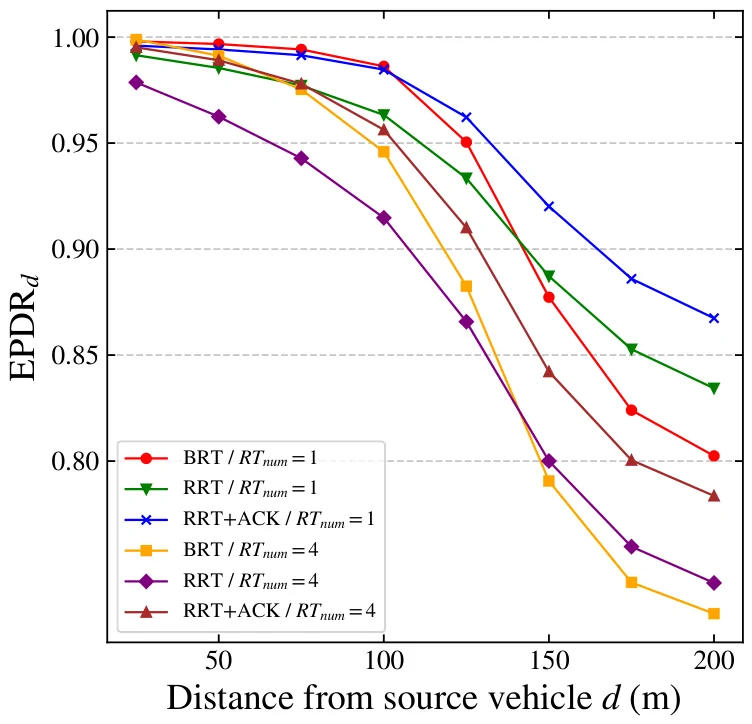
${\mathrm{EPDR}}_{d}$ with 80 vehicles and fixed retransmission counts.

**Figure 4:**
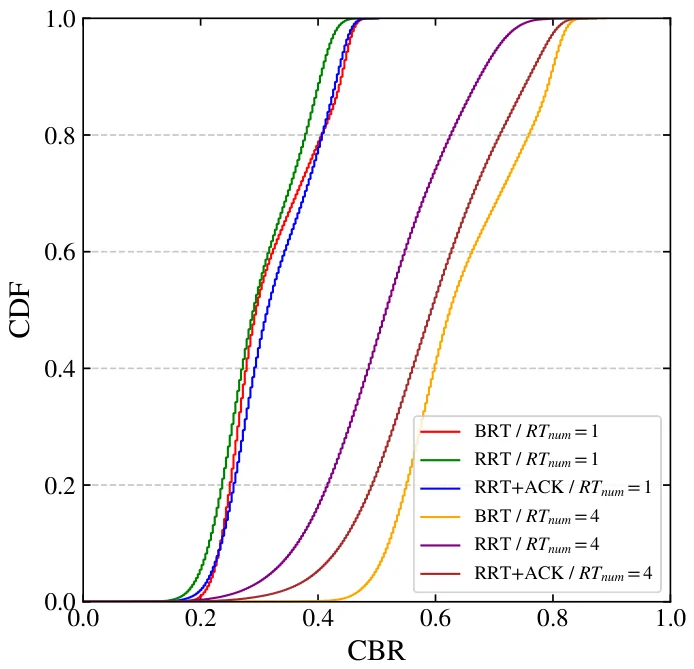
CBR cumulative distribution with 80 vehicles and fixed retransmission counts.

We next consider the high-density scenario with 80 vehicles. Figure 3 and Figure 4 respectively show ${\mathrm{EPDR}}_{d}$ and the CBR cumulative distribution for fixed retransmission counts. Table 3 summarizes the mean CBR.

With one retransmission, RRT + ACK achieves a higher ${\mathrm{EPDR}}_{d}$ than BRT for d>125m, consistent with the 40-vehicle result. With four retransmissions, however, ${\mathrm{EPDR}}_{d}$ decreases substantially for every scheme. Figure 4 and Table 3 show that the CBR distribution shifts toward higher values and that the mean CBR rises sharply. These results show that using a large fixed retransmission count raises channel load and lowers delivery rate in this scenario, indicating a stronger contention penalty from additional transmissions. In high-density environments, the retransmission count must therefore be adapted to the congestion state.

### 5.4. Evaluation of Dynamic Control

**Figure 5:**
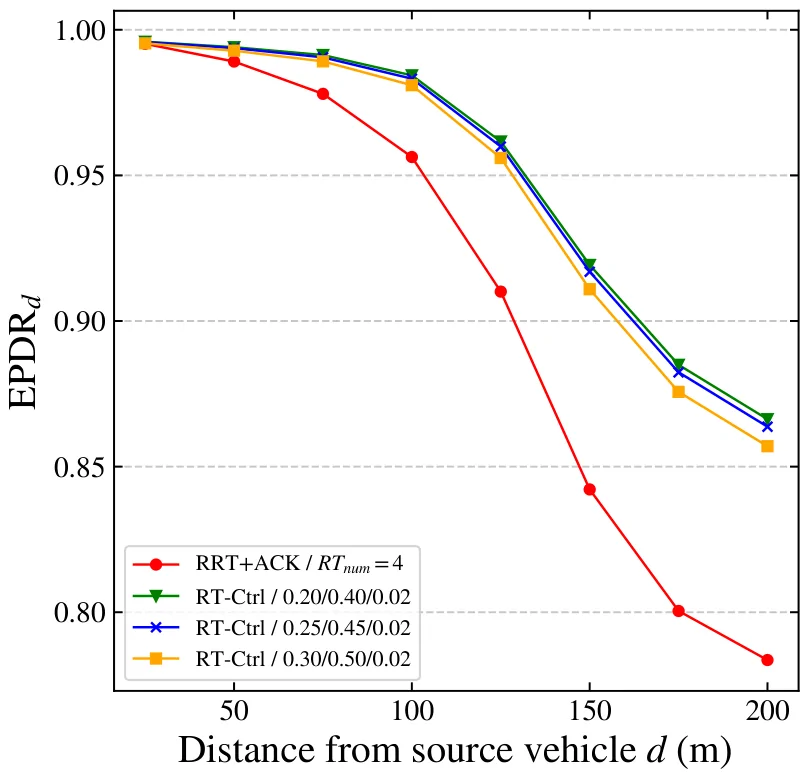
${\mathrm{EPDR}}_{d}$ with RT-Ctrl. RRT + ACK uses a fixed ${\mathrm{RT}}_{\mathrm{num}}=4$; RT-Ctrl uses ${\mathrm{RT}}_{\max}=4$, $K_{\min}=1$, and the three tested $\left(T_{\mathrm{target}},T_{\max},H\right)$ triples.

**Figure 6:**
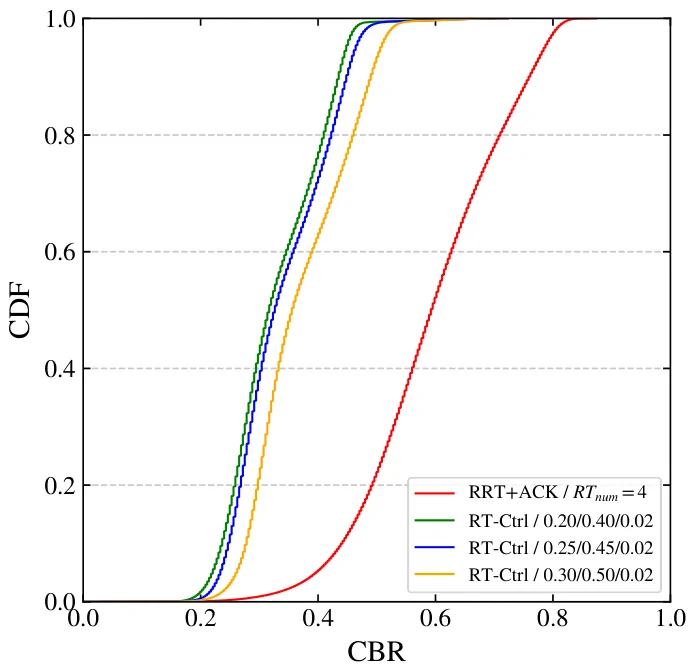
CBR cumulative distribution with RT-Ctrl. RRT + ACK uses a fixed ${\mathrm{RT}}_{\mathrm{num}}=4$; RT-Ctrl uses ${\mathrm{RT}}_{\max}=4$, $K_{\min}=1$, and the three tested $\left(T_{\mathrm{target}},T_{\max},H\right)$ triples.

Figure 5 and Figure 6 show ${\mathrm{EPDR}}_{d}$ as a function of distance *d* and the cumulative distribution of the CBR observed by the source at the initial transmission of each packet, respectively.

Introducing CBR-based retransmission-count control markedly improves ${\mathrm{EPDR}}_{d}$ compared with RRT + ACK using a fixed ${\mathrm{RT}}_{\mathrm{num}}=4$. Here, ${\mathrm{RT}}_{\max}=4$ is an upper bound and does not mean that every vehicle always retransmits four times. The best tested setting, $\left(T_{\mathrm{target}},T_{\max},H\right)=\left(0.20,0.40,0.02\right)$, increases ${\mathrm{EPDR}}_{200m}$ from 0.7836 to 0.8663 (with an improvement of approximately 10.6%) and decreases the mean CBR from 0.5945 to 0.3289 (with an improvement of approximately 44.7%). Its mean retransmission count is 1.030. The settings (0.25,0.45,0.02) and (0.30,0.50,0.02) yield ${\mathrm{EPDR}}_{200m}$ values of 0.8637 and 0.8570, respectively. These results show that suppressing excessive retransmissions under congestion reduces contention while retaining at least one retransmission for reliability.

Table 4 summarizes the data underlying Figure 5 and Figure 6, which use a minimum of one retransmission. RT-A, RT-B, and RT-C denote the parameter triples (0.20,0.40,0.02), (0.25,0.45,0.02), and (0.30,0.50,0.02), respectively. The results for the fixed-four-retransmission baselines are the same as those in Table 3.

### 5.5. Discussion

For the minimum-one-retransmission result, RT-A has EPDR 0.8663 and CBR 0.3289, close to the fixed-one RRT + ACK values of 0.8674 and 0.3260. Its average retransmission count is 1.0297. Thus, RT-A successfully avoids overloading the channel; these observations support avoidance of the excessive load of four fixed retransmissions. However, this result does not demonstrate superiority over a method with a well-chosen fixed retransmission count and a method adaptive to time-varying congestion.

The overall transmission counts per 80-vehicle run are 389,898.4 for RRT + ACK (four fixed retransmissionretransmissions), 159,989.4 for RT-A, and 157,614.3 for RRT + ACK (one fixed retransmission). Compared with RRT + ACK (four fixed retransmissionretransmissions), RT-A reduces transmissions by 58.97% and relay data transmissions by approximately 72.1%. Together with the EPDR and CBR results, these counts support the interpretation that suppressing excessive retransmissions improves the tradeoff between delivery rate and channel load in this scenario. The counters include source and relay data transmissions but exclude ACK transmissions, so they do not measure total control-inclusive resource use. RT-A is the best-performing setting among the three tested parameter triples, but not a universally optimal configuration.

The results suggest two distinct mechanisms. Relaying can provide a propagation path that differs from the obstructed direct path, whereas the ACK enables the source to retain retransmission responsibility when the selected relay has not received the packet. These benefits are conditional on the ideal-feedback assumption. Increasing the fixed retransmission count, in contrast, adds load to the shared resource pool and can offset the benefit of additional reception opportunities. RT-Ctrl addresses this trade-offtradeoff by limiting repeated transmissions as the observed CBR increases.

Session-level control limits how often the retransmission budget changes, while probabilistic count selection allows different vehicles to use different counts under similar CBR conditions. The corresponding trade-offtradeoff is responsiveness: a count retained until the next reselection event can lag behind changing traffic or topology. Under severe congestion, RT-Ctrl allows $K_{\mathrm{retx}}=0$, in which case only the initial transmission is performed. CBR also measures aggregate activity rather than the marginal reception benefit of a particular relay transmission, and it does not strictly enforce the future occupancy of each vehicle. Combining the method with CR-limit compliance or transmission-interval control remains a subject for future work [11,12].

The engineering value of the scheme must be considered jointly in terms of delivery rate and resource use. The approximately 8.6% improvement concerns the basic relay mechanism, whereas the additional contribution of RT-Ctrl is the reduction of excessive retransmission traffic under congestion. RRT + ACK includes the initial source transmission, source retransmissions before responsibility is transferred, and relay retransmissions thereafter. A larger fixed count increases the potential traffic from this sequence. Accordingly, the measured delivery benefit should be weighed against coordination signaling, information maintenance, and processing requirements.

Our APCC 2024 study reported delivery ratio as a function of elapsed time for the basic relay scheme with 40 vehicles, one retransmission, and ideal ACKs [9]. Those results do not establish deadline performance for RT-Ctrl under the present higher-density conditions or quantify practical ACK delay. The intended use is periodic dissemination of local vehicle-state information around obstructed intersections. Reusing the resource reserved by the source avoids an additional resource-selection procedure at the relay, but a receiver served through the relay still has to wait for a usable relay opportunity. Practical processing and feedback delay can increase that wait. The 100ms packet-generation interval is a traffic setting, typically used for local dissemination of vehicle information. Separately, the EPDR implementation counts only receptions with delay below 100ms, so the reported EPDR characterizes delivery within that evaluation window (deadline) under ideal feedback. Applicability to a particular service therefore requires comparing its deadline with the evaluation window and assessing the missing -feedback and delay impacts.

Directional LQIs summarize observed reception history; they do not guarantee that the relay maximizing the estimated delivery ratio remains best as vehicles move. Faster information refresh can reduce staleness but also increases signaling or processing requirements. For a direct evaluation of the selection equations over *M* relay candidates and *N* neighboring receivers, the computational cost is O(MN), assuming constant-cost evaluation of each link term for the bounded retransmission count. Directional aggregation reduces advertised information, not necessarily the full selection complexity. The count controller itself uses a fixed number of threshold operations, and a straightforward implementation of its binomial sampling uses at most $K_{\max}$ Bernoulli trials. These analytical observations are not execution-time measurements. The evaluation covers 40 and 80 vehicles at 54–72km/h with one selected relay, and does not establish a maximum supported density, speed, or relay-chain length.

For four directions and a hypothetical encoding of *b* bits per LQI value, the directional value fields would occupy 4b bits per advertisement, excluding identifiers, headers, and other coordination information. This illustrates the value-field scaling only; it is not a measured control-packet size. A full overhead budget must also include the advertisement rate, relay identification, reservation coordination, and feedback resources. The present transmission-count results quantify data traffic; future work will extend the analysis to include LQI signaling, coordination messages, and physical ACK resources.

An ACK lost at the source can leave it retransmitting even though the relay has decoded the packet; if the relay starts forwarding independently, their actions may conflict. Delayed ACKs can also arrive after a planned retransmission opportunity. A practical protocol must define responsibility transfer, handling of late ACKs, and fallback behavior, and account for any additional signaling. The present results characterize ideal-feedback operation. Future work will evaluate ACK loss and feedback delay, including their impacts on responsibility transfer, delivery reliability, and resource use.

## 6. Conclusions

This paper extended RRT + ACK, a relay-based broadcast scheme previously reported at APCC 2024, with CBR-based retransmission-count control. Directional LQI aggregation and ACK-assisted transfer of retransmission responsibility are the inherited components; the additional contribution is their combination with session-level control of retransmission traffic and the expanded congestion evaluation. Under the ideal error-free ACK assumption, the evaluation showed that the basic RRT + ACK scheme improves ${\mathrm{EPDR}}_{200m}$ by approximately 8.6% compared with conventional blind retransmission.

The high-density evaluation showed that a fixed retransmission count aggravates channel congestion. Under the best tested RT-Ctrl parameter setting, CBR-based retransmission-count control improves ${\mathrm{EPDR}}_{200m}$ from 0.784 to 0.866 by approximately 10.6% and reduces the mean CBR from 0.595 to 0.329 by approximately 44.7% relative to RRT + ACK.

Future work will evaluate ACK loss and delay, quantify control-inclusive resource overhead and delivery deadline, and compare the scheme with existing adaptive retransmission methods under common simulation conditions. It will also examine larger or differently shaped road networks, directional partitions and map-assisted relay selection, and integration of MCS and Resource Reservation Interval (RRI) control into a broader congestion-control framework. 

## Figures and Tables

**Table 1:** Simulation conditions.

Item	Value
Simulator	Scenargie with NR-V2X extensions
Scenario	300m×300m intersection
Number of vehicles	40 or 80
Vehicle speed	54–72km/h
Carrier frequency	5.9GHz
Bandwidth	10MHz
Slot duration	1ms
Packet size	256 bytes
Transmission interval	100ms
Radio propagation model	ITU-R P.1411
Fading model	Nakagami
Number of retransmissions	1, 4, or dynamically controlled
Simulation duration	120s
Number of simulation runs	20 runs with different seeds

**Table 2:** Run-level variability: Figure 2 (ratios).

Scheme/Setting	Metric	n	Mean	SD	95% CI
BRT R = 1	EPDR200m	20	0.8190	0.0028	[0.8177, 0.8203]
BRT R = 1	Mean CBR	20	0.1619	0.0011	[0.1614, 0.1624]
RRT R = 1	EPDR200m	20	0.8713	0.0027	[0.8700, 0.8725]
RRT R = 1	Mean CBR	20	0.1597	0.0008	[0.1593, 0.1601]
RRT + ACK R = 1	EPDR200m	20	0.8899	0.0021	[0.8889, 0.8908]
RRT + ACK R = 1	Mean CBR	20	0.1677	0.0010	[0.1672, 0.1681]

**Table 3:** Run-level variability: Figure 3 and Figure 4 (ratios).

Scheme/Setting	Metric	n	Mean	SD	95% CI
BRT R = 1	EPDR200m	20	0.8025	0.0015	[0.8017, 0.8032]
BRT R = 1	Mean CBR	20	0.3188	0.0010	[0.3183, 0.3192]
RRT R = 1	EPDR200m	20	0.8343	0.0023	[0.8333, 0.8354]
RRT R = 1	Mean CBR	20	0.3018	0.0014	[0.3011, 0.3024]
RRT + ACK R = 1	EPDR200m	20	0.8674	0.0018	[0.8666, 0.8682]
RRT + ACK R = 1	Mean CBR	20	0.3260	0.0013	[0.3254, 0.3266]
BRT R = 4	EPDR200m	20	0.7280	0.0017	[0.7272, 0.7288]
BRT R = 4	Mean CBR	20	0.6439	0.0013	[0.6433, 0.6445]
RRT R = 4	EPDR200m	20	0.7425	0.0017	[0.7417, 0.7433]
RRT R = 4	Mean CBR	20	0.5169	0.0019	[0.5160, 0.5177]
RRT + ACK R = 4	EPDR200m	20	0.7836	0.0025	[0.7824, 0.7848]
RRT + ACK R = 4	Mean CBR	20	0.5945	0.0016	[0.5938, 0.5953]

**Table 4:** Run-level variability: Figure 5 and Figure 6 (RT-Ctrl $K_{\min}=1$; ratios).

Scheme/Setting	Metric	n	Mean	SD	95% CI
RRT + ACK R = 4	EPDR200m	20	0.7836	0.0025	[0.7824, 0.7848]
RRT + ACK R = 4	Mean CBR	20	0.5945	0.0016	[0.5938, 0.5953]
RT-A	EPDR200m	20	0.8663	0.0019	[0.8654, 0.8672]
RT-A	Mean CBR	20	0.3289	0.0013	[0.3283, 0.3295]
RT-B	EPDR200m	20	0.8637	0.0020	[0.8628, 0.8647]
RT-B	Mean CBR	20	0.3406	0.0027	[0.3393, 0.3418]
RT-C	EPDR200m	20	0.8570	0.0024	[0.8559, 0.8581]
RT-C	Mean CBR	20	0.3727	0.0015	[0.3720, 0.3734]

## Data Availability

The data presented in this study are available from the corresponding author upon reasonable request.

## Abbreviations

The following abbreviations are used in this manuscript:

ACK	Acknowledgment
BRT	Blind Retransmission
CBR	Channel Busy Ratio
CR	Channel Occupancy Ratio
EPDR	Evaluation Packet Delivery Ratio
HARQ	Hybrid Automatic Repeat reQuest
LQI	Link Quality Indicator
MCS	Modulation and Coding Scheme
NLOS	Non-Line-of-Sight
NR-V2X	New Radio Vehicle-to-Everything
PDR	Packet Delivery Ratio
PRR	Packet Reception Ratio
PSCCH	Physical Sidelink Control Channel
PSFCH	Physical Sidelink Feedback Channel
PSSCH	Physical Sidelink Shared Channel
RRI	Resource Reservation Interval
RRT	Relay-assisted Retransmission
RT-Ctrl	Retransmission-count Control
SCI	Sidelink Control Information
SPS	Semi-Persistent Scheduling

## Appendix A. Numerical Values Corresponding to Figure 2, Figure 3, Figure 4, Figure 5 and Figure 6

All entries are dimensionless ratios except distance. The EPDR tables reproduce the plotted packet-pooled means, rounded to four decimal places. CBR tables give selected values of F(x)=P(CBR≤x) from the pooled observations; they are descriptive summaries, not CDF confidence intervals. RT-A, RT-B, and RT-C denote $\left(T_{\mathrm{target}},T_{\max},H\right)\;=\;\left(0.20,0.40,0.02\right)$, (0.25,0.45,0.02), and (0.30,0.50,0.02).

**Table A1:** All EPDR plot values for Figure 2.

d (m)	BRT R = 1	RRT R = 1	RRT + ACK R = 1
25	0.9982	0.9932	0.9967
50	0.9978	0.9906	0.9960
75	0.9969	0.9870	0.9951
100	0.9925	0.9796	0.9916
125	0.9632	0.9586	0.9752
150	0.8953	0.9206	0.9396
175	0.8415	0.8889	0.9078
200	0.8190	0.8713	0.8899

**Table A2:** All EPDR plot values for Figure 3.

d (m)	BRT R = 1	RRT R = 1	RRT + ACK R = 1	BRT R = 4	RRT R = 4	RRT + ACK R = 4
25	0.9980	0.9915	0.9960	0.9989	0.9787	0.9952
50	0.9967	0.9855	0.9942	0.9913	0.9625	0.9891
75	0.9942	0.9772	0.9915	0.9753	0.9429	0.9780
100	0.9862	0.9633	0.9847	0.9459	0.9148	0.9563
125	0.9505	0.9335	0.9622	0.8825	0.8658	0.9101
150	0.8773	0.8872	0.9202	0.7906	0.8001	0.8422
175	0.8240	0.8528	0.8860	0.7427	0.7596	0.8004
200	0.8025	0.8343	0.8674	0.7280	0.7425	0.7836

**Table A3:** Selected CBR CDF values for Figure 4.

Scheme/Setting	F(0.20)	F(0.30)	F(0.40)	F(0.50)	F(0.60)	F(0.70)	F(0.80)
BRT R = 1	0.0092	0.5218	0.7890	1.0000	1.0000	1.0000	1.0000
RRT R = 1	0.0548	0.5393	0.8891	1.0000	1.0000	1.0000	1.0000
RRT + ACK R = 1	0.0199	0.4347	0.7792	1.0000	1.0000	1.0000	1.0000
BRT R = 4	0.0006	0.0008	0.0014	0.0565	0.4077	0.6765	0.9203
RRT R = 4	0.0033	0.0337	0.1669	0.4497	0.7410	0.9399	0.9994
RRT + ACK R = 4	0.0010	0.0088	0.0544	0.2223	0.5214	0.7786	0.9760

**Table A4:** All EPDR plot values for Figure 5.

d (m)	RRT + ACK R = 4	RT-A	RT-B	RT-C
25	0.9952	0.9959	0.9958	0.9954
50	0.9891	0.9940	0.9937	0.9928
75	0.9780	0.9913	0.9905	0.9892
100	0.9563	0.9844	0.9832	0.9810
125	0.9101	0.9616	0.9599	0.9560
150	0.8422	0.9193	0.9169	0.9109
175	0.8004	0.8850	0.8824	0.8757
200	0.7836	0.8663	0.8637	0.8570

**Table A5:** Selected CBR CDF values for Figure 6.

Scheme/Setting	F(0.20)	F(0.30)	F(0.40)	F(0.50)	F(0.60)	F(0.70)	F(0.80)
RRT + ACK R = 4	0.0010	0.0088	0.0544	0.2223	0.5214	0.7786	0.9760
RT-A	0.0167	0.4244	0.7707	0.9943	0.9976	1.0000	1.0000
RT-B	0.0057	0.3790	0.7283	0.9916	0.9970	1.0000	1.0000
RT-C	0.0027	0.2105	0.6315	0.9337	0.9966	0.9999	1.0000
